# The effect of mesenchymal stem cell transplantation on the recovery of bladder and hindlimb function after spinal cord contusion in rats

**DOI:** 10.1186/1471-2202-11-119

**Published:** 2010-09-16

**Authors:** Won Beom Park, Soo Yeon Kim, Sang Hoon Lee, Hae-Won Kim, Jeong-Soo Park, Jung Keun Hyun

**Affiliations:** 1 Department of Rehabilitation Medicine, College of Medicine, Dankook University, Cheonan, Korea; 2 Department of Nanobiomedical Science & WCU Nanobiomedical Research Center, Dankook University, Cheonan, Korea; 3 Department of Biomedical Engineering, Korea University, Seoul, Korea; 4 Department of Biomaterials Science, School of Dentistry, Dankook University, Cheonan, Korea; 5 Department of Biochemistry, College of Medicine, Dankook University, Cheonan, Korea; 6 Institute of Tissue Regeneration Engineering (ITREN), Dankook University, Cheonan, Korea

## Abstract

**Background:**

Mesenchymal stem cells are widely used for transplantation into the injured spinal cord in vivo model and for safety, many human clinical trials are continuing to promote improvements of motor and sensory functions after spinal cord injury. Yet the exact mechanism for these improvements remains undefined. Neurogenic bladder following spinal cord injury is the main problem decreasing the quality of life for patients with spinal cord injury, but there are no clear data using stem cell transplantation for the improvement of neurogenic bladder for in vivo studies and the clinical setting.

The purpose of this study was to delineate the effect of human mesenchymal stem cell (hMSCs) transplantation on the restoration of neurogenic bladder and impaired hindlimb function after spinal cord contusion of rats and the relationship between neurotrophic factors such as brain derived neurotrophic factor (BDNF) and neurotrophin-3 (NT-3) and bladder and hindlimb functions.

**Results:**

Modified moderate contusion injury were performed on the thoracic spinal cord of Sprague-Dawley rats using MASCIS impactor and hMSCs, human fibroblasts or phosphate-buffered saline were transplanted into injured spinal cord 9 days after injury for hMSC and two control groups respectively. Ladder test showed more rapid restoration of hindlimb function in hMSC group than in control group, but Basso, Beattie, and Bresnahan score and coupling score were not different significantly among hMSC and two control groups. Neurogenic bladder was not improved in either group. ED1 positive macrophages were significantly reduced in hMSC group than in two control groups, but ELISA and RT-PCR studies revealed BDNF and NT-3 levels in spinal cord and bladder were not different among hMSC and two control groups regardless the experimental duration.

**Conclusion:**

hMSC transplantation was effective in reducing inflammatory reaction after spinal cord contusion of rats but not sufficient to recover locomotor and bladder dysfunction. BDNF and NT-3 levels in the spinal cord and bladder were not increased 28 and 56 days after hMSC transplantation.

## Background

Neurogenic bladder following spinal cord injury as well as paralysis is a major medical problem that has social implications due to quality of life issues [1]. Urogenital diseases including urinary tract infection is a major cause of morbidity and mortality although many complications are now decreasing with improved management [2]. The present goals of the management of neurogenic bladder in patients with spinal cord injury are the preservation of the renal function and increasing the quality of life for patients by minimizing complications [1], but the restoration of bladder function after spinal cord injury has proven to be difficult to achieve because techniques in axonal regeneration and tissue repair remain quite limited until now.

Recently research efforts have focused on attempts to regenerate the injured spinal cord using neurotrophic factors and drug delivery systems [3], biomaterials [4] and cell transplantation [5]. Stem cell transplantation is one of the most promising fields for spinal cord regeneration because stem cells can achieve fundamental regeneration of injured spinal cord by replacing damaged neuronal tissues [6] and it has the potential to be combined with various biomaterials for co-transplantation with neurotrophic factors [7-9]. Mesenchymal stem cells have potential for various therapeutic applications and are clinically attractive for spinal cord repair since they can be obtained easily from adult bone marrow, blood or adipose tissue cells. As well, autograft transplantation does not induce immune rejection. Several in vivo studies revealed that MSCs transplanted into the central nervous system can be transdifferentiated into astrocytes and neurons as well as tissues from mesodermal origin [10,11], and are effective in the partial recovery of locomotor function after damage to the central nervous system [12]. Clinical trials of autologous hMSC transplantation were performed on acute and chronic patients with spinal cord injury [13-16] and the safety and some clinical improvements for human were reported but the exact mechanism of hMSCs on the functional recovery are still unclear [17].

Endogenous neurotrophic factors in the spinal cord and bladder are considered to promote neuronal survival and axonal growth after spinal cord injury [18,19] and the administration of exogenous brain-derived neurotrophic factor (BDNF) and neurotrophin-3 (NT-3) has been reported to contribute to the regeneration of damaged neuronal cells, improvement of paralyzed hindlimb function as well as neurogenic bladder after spinal cord injury [20,21].

Nevertheless there is no report revealing the relationship between the recovery of locomotor function or neurogenic bladder after hMSC transplantation and the status of endogenous neurotrophic factors in the spinal cord or bladder in spinal cord injury models. Therefore the purpose of these experiments was to reveal the effects of hMSC transplantation on the recovery of neurogenic bladder and locomotor function in animal models of spinal cord injury and investigate the relationship between these results and the level of endogenous neurotrophic factors in the spinal cord and bladder.

## Results

### Hindlimb functions

Basso, Beattie, and Bresnahan (BBB) scale, coupling score and ladder score showed gradual restoration in all hMSC and two control groups throughout the 56 days following spinal cord injury. Repeated measure ANOVA revealed that there was no difference between the time groups sacrificed at 28 days and 56 days following transplantation (PTD28 and PTD56) until 28 days following transplantation within the same transplantation group (p > 0.05).

**Figure 1:**
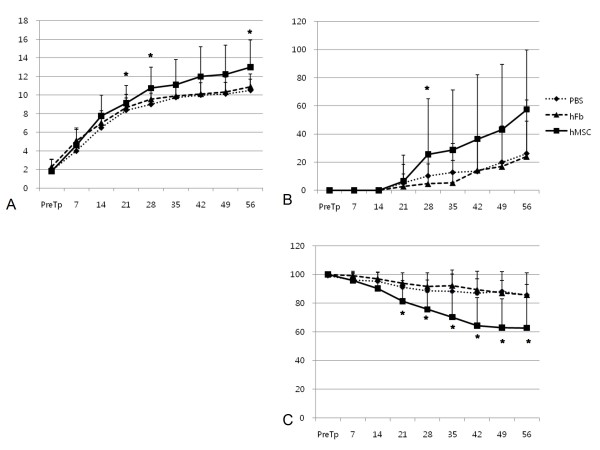
**Locomotor function in experimental (hMSC) and two control (PBS and hFb) groups**. (A) BBB (Basso, Beattie, Bresnahan) scores, (B) coupling scores and (C) ladder scores showed gradual restoration in experimental and control groups, but all three scales of experimental group (hMSC) at some periods (asterisks) were restored more than those of control group (hFb). PTD: post-transplantation day, PBS: control group received phosphate-buffered saline, hFb: control group received human fibroblast, hMSC: experimental group received human mesenchymal stem cell, *p < 0.05 by one-way ANOVA with Bonferroni post hoc test.

BBB scale of hMSC group were higher than those of two control groups which received phosphate-buffered saline (PBS) or human fibroblasts (hFbs) at post-transplantation day (PTD) 21, 28 and 56 (Fig. 1A, p < 0.05), and coupling score of hMSC group were higher than those of two control groups only at PTD28 (Fig. 1B, p < 0.05). Ladder score in all experimental periods exceeding PTD14 revealed that the erroneous step rate of hMSC group was reduced more than those of two control groups (Fig. 1C, p < 0.05).

### Bladder volume and urodynamic study

Urodynamic study at PTD26 and PTD56 revealed that all the bladder in hMSC and two control groups was hyperreflexic type (67-83% within each case), and there was no difference of micturition frequency and pressure, the chance of detrusor contraction without micturition, and bladder volume among hMSC group and two control groups (Table 1).

**Table 1:** Urodynamic Study and Bladder Volume of Experimental and Control Groups at 28 and 56 Days after Transplantation.

	PTD28			PTD56			p-value
		
	PBS (n = 7)	hFb (n = 11)	hMSC (n = 9)	PBS (n = 7)	hFb (n = 9)	hMSC (n = 6)	
Voiding frequency (time/min)	0.80 ± 0.09	0.82 ± 0.16	0.76 ± 0.32	0.79 ± 0.11	0.82 ± 0.19	0.92 ± 0.07	>0.05*
Maximal pressure (cmH_2_O)	9.21 ± 5.30	9.60 ± 4.2	9.70 ± 4.11	9.00 ± 5.77	8.11 ± 7.39	10.33 ± 4.50	>0.05*
Pattern of regularity (%)†	43	45	44	43	22	50	>0.05*
Detrusor contraction without voiding (%)†	43	36	44	43	44	50	>0.05*
Gross pattern of voiding (%)†							>0.05*
Flaccid	29	27	13	29	33	17	
Hyperreflexia	71	73	87	71	67	83	
Normal	0	0	0	0	0	0	
Bladder volume (mm*)	1345.25 ± 1206.27	1263.75 ± 838.05	1279.57 ± 1310.69	1874.47 ± 1452.13	1925.99 ± 1591.44	1594.45 ± 703.36	>0.05*

PTD: post-transplantation day, PBS: control group received phosphate-buffered saline, hFb: control group received human fibroblast, hMSC: experimental group received human mesenchymal stem cell * by one-way AVOVA, ** by Fisher's exact test, † No. of detectable cases (%)

### ELISA study

**Figure 2:**
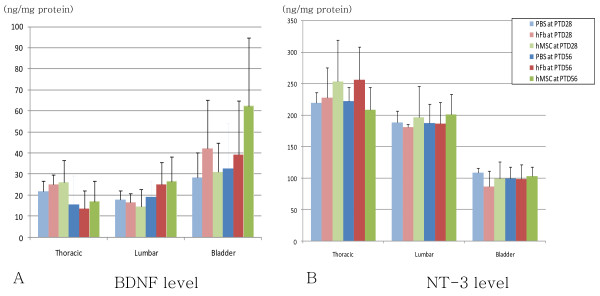
**mRNA levels of neurotrophic factors by ELISA in experimental (hMSC) and two control (PBS and hFb) groups at 28 and 56 days after transplantation**. (A) BDNF level and (B) NT-3 level in the thoracic and lumbar spinal cords and bladder showed no statistical difference among experimental and two control groups at PTD28 and PTD56. hMSC: experimental group received human mesenchymal stem cell, PBS: control group received phosphate-buffered saline, hFb: control group received human fibroblast, PTD: post-transplantation day

When comparing the protein levels of BDNF and NT-3 among two control groups and hMSC group, there was no significant difference of the concentration of BDNF and NT-3 in thoracic and lumbar spinal cords and bladder between hMSC and control groups at PTD28 and PTD56 (Fig. 2).

### RT-PCR

**Figure 3:**
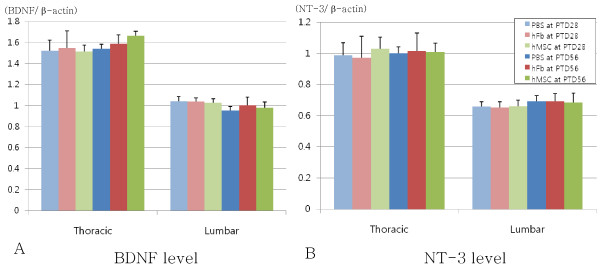
**Protein levels of neurotrophic factors by RT-PCR in experimental (hMSC) and two control (PBS and hFb) groups at 28 and 56 days after transplantation**. (A) BDNF level and (B) NT-3 level in the thoracic and lumbar spinal cords also showed no statistical difference among experimental and two control groups at PTD28 and PTD56. hMSC: experimental group received human mesenchymal stem cell, PBS: control group received phosphate-buffered saline, hFb: control group received human fibroblast, PTD: post-transplantation day

RT-PCR was performed to injured thoracic spinal cord and lumbar spinal cord, and the levels of BDNF or NT-3/β-actin were not different among two control groups and hMSC group regardless of experimental periods (PTD28 or PTD56) (Fig. 3).

### Immunohistochemistry

**Figure 4:**
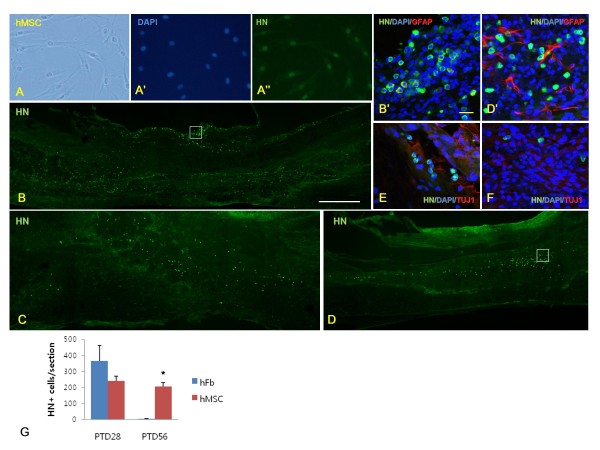
**Immunohistochemistry of human mesenchymal stem cells (hMSCs) in vitro and in vivo studies**. (A) hMSCs were well stained by anti-human nucleus antibody (HN) in vitro (A"), and injured area of thoracic spinal cord after transplantation of human fibroblasts (hFbs) at PTD28 (B) and after transplantation of hMSCs at PTD28 (C) and PTD56 (D) was stained with HN. B' and D' images are magnified images of B and D white-lined boxes respectively. Double staining with HN (green color) and anti-GFAP antibody (red color) (B' and D'), and HN (green color) and anti-beta III tubulin antibody (red color) after hMSC transplantation at PTD28 (E) and PTD56 (F) indicated that transplanted hMSCs into injured spinal cord did not differentiated into neurons nor astrocytes 28 and 56 days after transplantation. (G) Total number of HN-positive cells per section was higher in hMSC group than in hFb group (asterisk) at PTD56. White scale bar in B = 500 μm, yellow scale bar in B' = 20 μm.

We found that hMSCs were well stained with anti-human nucleus antibody (HN) in vitro (Fig. 4A) and HN-positive cells existed within the injured thoracic spinal cord of hFb group at PTD28 (Fig. 4B) and hMSC group at PTD28 (Fig. 4C) and PTD56 (Fig. 4D). But HN and anti-GFAP antibody (Fig. 4D') or anti-beta III tubulin antibody at PTD28 (Fig. 4E) and PTD56 (Fig. 4F) in hMSC group were not double-stained, thus transplanted hMSCs into injured spinal cord did not differentiated into neurons nor astrocytes 28 and 56 days after transplantation. Fifty six days after transplantation, total number of HN-positive cells per section was higher in hMSC group than in hFb group at PTD56 (Fig. 4G).

**Figure 5:**
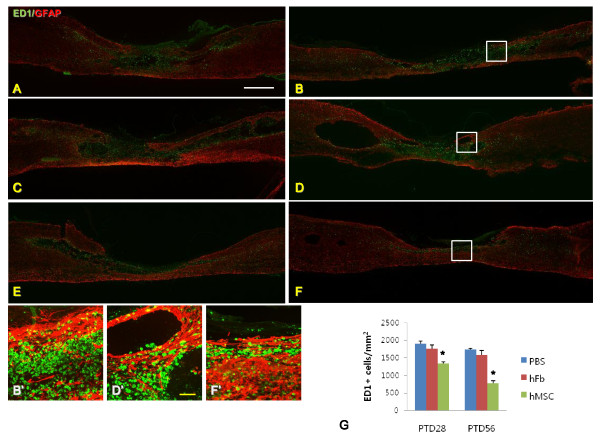
**Injured spinal cords of PBS, hFB and hMSC groups at PTD28 and PTD56 stained with anti-macrophages/monocytes (ED1) antibody (green color) and anti-GFAP antibody (red color; A-F)**. PBS group at PTD28 (A) and PTD56 (B), and hFB group at PTD28 (C) and PTD56 (D) showed many macropahges and monocytes were existed within the injured spinal cords compared with hMSC group at PTD28 (E) and PTD56 (F). . B', D' and F' images are magnified images of B, D and F white-lined boxes respectively. The number of macrophages and monocytes was lower in hMSC group than in PBS and hFb groups at PTD28 and PTD56 (asterisk, G). White scale bar in A = 1 mm, yellow scale bar in D' = 100 μm.

We checked the boundary of host tissue surrounding the injured area indirectly by GFAP staining showing host astrocytes and macrophages by ED1 staining in the sagittal section of epicenter area (Fig. 5). PBS and hFb groups showed larger cavities and fewer GFAP-positive areas than hMSC group in the injured area at PTD28 and PTD56 (Fig. 5A-F). ED1 positive macrophages were significantly fewer near the center of injured spinal cord in hMSC group (Fig. 5G) than in PBS and hFb groups.

## Discussion

Neuronal cells within the injured area are directly damaged just after trauma in the spinal cord and the secondary injury, caused by the inflammatory process through activations of macrophages, neutrophils and lymphocytes, leads to cavity and scar formation which usually exaggerates the damage [22]. Clinical management for reducing the amount of cellular damage from secondary injury consists of steroid application within 8 hours after spinal cord injury. Steroids are the only class of drug which has been tested for clinical use in spinal cord injury and widely used in treatment of such cases [23], but it also has some detrimental effects including the risk for infections and gastrointestinal complications [24]. Inflammation process following spinal cord injury has both beneficial and detrimental effects [25] but several in vivo studies revealed that the inhibition of inflammation promoted improvements in motor and sensory functions [26,27]. We found that macrophages in the injured spinal cord area were significantly less in hMSC group than in control group, therefore transplanted mesenchymal stem cells may act to control the inflammatory process following spinal cord injury.

Stem cells such as embryonic stem cells, neural stem cells and MSCs were investigated in injured spinal cord regeneration [22]. MSCs were the first type of stem cells used to treat patients [28] and the only one stem cells whose safety has been established until recently [17]. The mechanism of MSC for the functional improvement following spinal cord injury is not clear but several possible explanations have suggested. MSCs can be transdifferentiated into neurons and glial cells [10], and they are able to act as a physical guidance for neurofilament outgrowth in the injured spinal cord [29]. But the functional properties of transdifferentiated neurons are still controversial in vitro [30,31] and in vivo studies [32]. We could not observe that any neurons or glial cells differentiated from hMSCs existed within the injured area of the spinal cord in our study. The reduction of inflammatory process within the injured spinal cord resulting in the consequential decrement of secondary injury upon hMSCs transplantation may be related with functional restoration following spinal cord injury, but we did not observe any significant improvement of locomotor and bladder function. The exact mechanism of how transplanted hMSCs affect inflammatory reaction following spinal cord injury is not clear however it is known that MSCs could reduce the inflammatory process in animal lung [33].

Wilkins et al. [34] found that hMSCs from bone marrow secreted BDNF and these cells also promoted neuronal survival in vitro, where similarly Crigler et al. [35] revealed hMSCs could express transcripts encoding BDNF and β-NGF but not NT-3 and NT-4 in vitro and promoted neurite outgrowth within dorsal root ganglion explants. Mahmood et al.[36] and Kim et al. [37] transplanted rat or human MSCs into an in vivo model of traumatic brain injury and found that expression of BDNF, NT-3 and/or nerve growth factor were increased. Kim et al. also found some functional improvements in the hMSCs transplanted group [37]. In our study, we checked the amount of BDNF and NT-3 mRNAs and proteins at 28 days and 56 days following hMSCs transplantation in the spinal cord and bladder and no change was observed following transplantation regardless of the experimental periods. We performed ELISA and RT-PCR at chronic stage of spinal cord injury (PTD28 and 56) but we were not able to ascertain whether the level changes occurred in acute or subacute stages. The level of BDNF and NT-3 in hMSC transplanted group did not differ from those in control group 29 days after transplantation in a previous study [37], and the increments of the amount of neurotrophic factors including BDNF within the injured spinal cord occurred within 2 to 5 days following transplantation [25]. The application of exogenous BDNF following spinal cord injury could enhance locomotor recovery in some studies [20,38], therefore limited recovery of locomotor function after hMSC transplantation may be related with unchanged level of BDNF in our study.

Previous in vivo studies evaluating the effect of hMSC transplantation into injured spinal cord have focused on the functional changes in motor and sensory impairments following transplantation [39,40], but they did not evaluate autonomic function including neurogenic bladder. Mitsui et al. found some improvements in the neurogenic bladder of rats following spinal cord injury after transplantation of neural stem cells or precursor cells [41,42], and Temeltas et al. also reported some improvements of lower urinary function after rat MSCs transplantation [43].

In our study, neurogenic bladder was not recovered in the hMSC group compared with control group until 56 days after transplantation. Bladder dysfunction following spinal cord injury was severe enough not to recover spontaneously in control group which hindlimb function however showed gradual spontaneous recovery throughout the 56 days following spinal cord injury. Therefore hMSCs alone were likely insufficient to restore bladder function in this study.

The level of neurotrophic factors may also have the possibility of affecting bladder function. Mitsui et al. [21] has made a modified moderate contusion model, the same model in our study, where BDNF and NT-3 secreting fibroblasts were transplanted into the spinal cord and found that the neurogenic bladder was partially improved showing decrement of detrusor pressure and hyperreflexia 56 days following transplantation. They revealed that exogenous BDNF and NT-3 play an important role in repairing neurogenic bladder following spinal cord injury of rats [21]. In our study, endogenous BDNF and NT-3 levels in the spinal cord and bladder were unchanged after hMSCs transplantation and these results may explain unrecovered neurogenic bladder.

Limitation of our experiment was that we did not check the BDNF and NT-3 mRNA and protein levels at acute or subacute stages as mentioned earlier. We found that stem cell transplantation was not sufficient to restore adequate function of motor, sensory and autonomic systems following spinal cord injury in this study, therefore other methods including biomaterials for control and differentiation of transplanted stem cells and directionality of regenerating axons, neurotrophic factors for the growth of neurons and glial cells, in addition to drug delivery system for effective transport of stem cells or neurotrophic factors, should be developed and utilized in combination for successful regeneration of the injured spinal cord.

## Conclusions

Our study showed that the transplantation of hMSCs into the injured spinal cord of the modified moderate contusion model could reduce inflammation following spinal cord injury. But locomotor improvement was not obvious and neurogenic bladder and mRNA and protein levels of BDNF and NT-3 were unchanged after stem cell transplantation.

## Methods

### Surgical procedures for a modified moderate contusion model of rats

All procedures were approved by Dankook University's Institutional Animal Care and Use Committee (DKU-10-013). Adult female Sprague-Dawley rats (n = 60, weight 230-250 g) aged 12 weeks received modified moderate contusion injuries to the thoracic spinal cord. All rats were housed individually in a thermo-hygrostat (23°C, 50% humidity) with food and water available ad libitum. Animals were anesthetized with isoflurane (Forane*, Choongwae Pharma, Seoul, Korea). The skin layer, subcutaneous layer, and muscle layer were incised, laminectomy was performed, and the T8-9 level of the spinal cord was exposed without any damage or compression to the surrounding dura mater.

Using a Multicenter Animal Spinal Cord Injury Study (MASCIS) impactor (Rutgers, the State University of New Jersey, Newark, NJ), a 10-gram rod was dropped from a vertical distance of 25 mm onto the T9 level of the exposed spinal cords, and was allowed to rest for 5 seconds before it was lifted, thus resulting in a modified moderate contusion model that is the equivalent of Mitsui's method [41]. This model results in more motor deficits than a standard moderate contusion model. After contusion the muscle, subcutaneous, and skin were sutured in anatomical layers.

All animals received intramuscular injection of 40 mg/kg cefotiam hydrochloride (Fontiam*, Hanmi Pharma, Seoul, Korea) for 3 days and intraperitoneal injection of normal saline (3 ml) just after surgery. Animals also received oral administration of 10 mg/kg acetaminophen syrup (Tylenol*, Janssen Pharmaceutica, Titusville, NJ) for 3 days in order to reduce neuropathic and postoperative pain. Bladder expression was performed two times per day, and was continued until the amount of expressed urine was less than 0.5 ml/day.

### Transplantation of human mesenchymal stem cells and human fibroblasts

Spinal cord injured rats divided into one experimental group and two control groups by the type of transplanted cells or solutions; hMSC, hFb and PBS groups. At 9 days after injury, hMSC group (n = 22) received human mesenchymal stem cells, and hFb group (n = 22) and PBS group (n = 16) received human fibroblasts or phosphate-buffered saline respectively. hMSCs (Lonza Walkersville, Inc., Walkersville, MD) were cultured in MSCGM™ BulleKit* (PT-3001; MSCBM™, MSCGM™ SingleQuots* Kit, Lonza Walkersville, Inc., Walkersville, MD), and human fibroblasts (hFbs) were originated from 9-year old male foreskin (Biochemistry Lab. in Dankook University, Cheonan, Korea) and cultured in DMEM (Dulbecco's Modified Eagle's Medium, Welgene, Daegu, Korea) with high glucose. Ten percent fetal bovine serum (Invitrogen Corp., Carlsbad, CA) was added to DMEM and antibiotics (penicillin-streptomycin, Invitrogen Corp., Carlsbad, CA) were used for the prevention of bacterial infection.

After inhalation anesthesia and re-exposure of the contusion site, rats were placed in the stereotaxic frame (Jungdo B&P Inc., Seoul, Korea). For hMSC and hFb groups, 5 μL of hMSCs or hFbs (3 × 10* cells) were transplanted into the injured spinal cord respectively via Hamilton syringe (Hamilton company, Reno, NV) connected to a syringe pump (KD Scientific Inc., Holliston, MA) for 5 minutes. PBS group received 5 μL of phosphate-buffered saline (Dulbecco's phosphate-buffered saline, Welgene, Daegu, Korea) at the injured spinal cord with the same method. The needle was removed 10 minutes after finishing transplantation and muscle, subcutaneous, and skin layers were closed in layers and bladder expression continued.

Cyclosporin A (Cipol Inj*, Chongkundang Pharmaceutical Corp, Seoul, Korea) was administered at 10 mg/kg/d subcutaneously beginning one day before transplantation and continuing daily for 2 weeks after transplantation to all experimental and control groups. After that, the oral formula (Cipol Soln*, Chongkundang Pharmaceutical Corp, Seoul, Korea) with the same quantity and concentration was administered daily for the duration of the study.

Rats in hMSC and two control groups were also divided into two groups according to the amount of time since the operation; PTD28 and PTD56.

### Hindlimb functions

We used three scales for the evaluation of locomotor function of paralyzed hindlimb after spinal cord injury; Basso, Beattie, and Bresnahan (BBB) scale, coupling score and horizontal ladder test.

The Basso, Beattie, and Bresnahan (BBB) scale of no hindlimb movement is 0, and that of normal hindlimb movement is 21 [44]. Rats were analyzed by two observers who were blinded to the treatment received by each rat and positioned across from each other to observe both sides of the rats during 4- minute walking in the open field (cylindrical-shaped acrylic box; 90 cm diameter, 15 cm high) with a smooth floor. In case of rats with incomplete coordination of the forelimb and hindlimb for which BBB score is between 10 and 14, we used the forelimb/hindlimb coupling score to add more detailed information about gait coordination [45]. The coupling score was calculated as the percent of the number of corrected couplings (the number of ipsilateral forelimb steps which was followed by hindlimb steps) divided by the total number of couplings in the context of a continuous gait (the number of steps in the sequence minus one) for at least six steps and five scenes. A coupling score below 10 was regarded as 0% for the BBB score, and a coupling score above 14 was regarded as a BBB score of 100%. These locomotor scales were examined within a transparent, cylindrical-shaped (90 cm diameter, 15 cm high) acrylic box with a smooth floor.

Horizontal ladder test was performed on a runway made of acryl walls (10 cm tall, 127 cm long, 8 cm wide between walls, 1 cm between rungs) [46]. All rats were trained to walk from left to right on a runway several times for adaptation before testing and then captured with a digital camcorder. The ladder score was calculated as below.

Ladder score=Erroneous steps of hind limb/total steps of hind limb×100(%)

The locomotor function of each group was examined every 7 days until sacrifice. All locomotor tests were recorded for at least 4 minutes with a digital camcorder for coupling score and ladder score and were interpreted by two observers who were blinded to the identity of the rats.

### Bladder volume and urodynamic study

Bladder volume measurement and urodynamic study were performed as previously described [47]. Briefly, rats were anesthetized and the bladder was exposed. The diameter of the bladder dome was measured for indirect evaluation of bladder volume. The bladder volume was calculated as an imaginary sphere as follows:

$$\text{Bladder volume}={\left(\text{Diameter of bladder dome}/2\right)}^{3}\times \pi \times 4/3.$$

After checking the diameter, a double lumen polyethylene catheter (PE-160 and PE-50, Clay-Adams, Parsippany, NJ) was inserted into the bladder dome and fixed with sutures. One lumen (PE50) was connected to the pressure transducer and amplified and recorded by polygraph (Grass polygraph model 7E, Quincy, MA), and another lumen (PE160) was connected to a syringe filled with normal saline and loaded in an infusion pump (Baxter, Deerfield, IL). The room temperature normal saline was infused to the bladder at a rate of 10 ml/hour at first, and the speed was modified to 5 ml/hour after the first void and concomitant stop for 30 minutes to stabilize micturition cycles. We recorded the detrusor pressure just after saline filling, and checked the timing and numbers of drops of voiding to reveal the coordination of the detrusor and the external urethral sphincter. During the recording procedure, the anesthetic vapor level was gradually reduced to 0.5% equally to all controls and experimental groups in order to minimize the effect of anesthesia on micturition behavior.

Through the urodynamic study, we checked the maximal micturition pressure (cmH_2_O) and frequency (time/minute), gross voiding patterns and regularity of micturition frequency and pressure. Gross voiding patterns were described as normal, flaccid, and hyperreflexia according to the micturition frequency. A flaccid pattern was defined as no visible detrusor contraction, and a hyperreflexic pattern showed a higher frequency of detrusor contraction than the mean of the controls plus two standard deviations. An irregular micturition pattern was defined as a pattern of frequency or maximal pressure that was too variable to count or calculate.

### ELISA Measurements

Following urodynamic study, rats (n = 6 in hMSC and hFb group and n = 4 in PBS group at each experimental period) were deeply anesthetized and the thoracic and lumbar spinal cords and bladder were removed. All tissues were frozen using liquid nitrogen and stored in a deep freezer at -70°C.

Extracted thoracic and lumbar spinal cords were hemisected in the midline of sagittal plane and divided into two pieces for the analysis of ELISA and RT-PCR. All tissues were homogenized in 100 mM Tris/HCl (PH 7.0) lysis buffer containing 1 M NaCl, 4 mM EDTA, 2% Triton X-100, 2% bovine serum albumin, 0.1% sodium azide, and protease inhibitor (P8340, Sigma, St. Louis, MO). Protein concentrations were calculated using the BCA protein assay (Pierce, Rockford, IL). Expression of BDNF and NT-3 was examined by ELISA Kit (ChemiKine* Sandwich ELISA Kit, Chemicon International Inc., Temecula, CA), and the results were obtained using a microplate reader (Model 680, Bio-Rad, Hercules, CA) at a wavelength of 450 nm.

### RNA extraction and RT-PCR

Spinal cord tissues (n = 6 in hMSC and hFb group and n = 4 in PBS group at each experimental period) were dissected and stored with the same method as in the ELISA study. RNA was extracted from dissected thoracic and lumbar spinal cords using Trizol reagent (Invitrogen Corp., Carlsbad, CA). The concentration of extracted RNA was measured with Nanodrop (Thermo Fisher Scientific Inc., Waltham, MA), and 5 ug of RNA was used for the process of reverse transcription. Total RNA was reverse transcribed using oligo-dT primer and SuperScript II transcriptase (Invitrogen Corp., Carlsbad, CA), and cDNA sequences for rat BDNF and NT-3 were obtained and PCR amplification of cDNA was performed. β-actin was used as control to estimate the amount of RNA analyzed. Primer sequence for rat BDNF, NT-3 and β-actin were as follows: BDNF (forward: 5'-CAGTGGACATGTCCGGTGGGACGGTC-3', reverse: 5'-GTTGTGGTTTGTTGCCGTTGCCAAGAA-3'), NT-3 (forward 5'-GCAACAGACACAGAACTACTA-3', reverse 5'-GCCTGTGGGTGACCGACAAGT-3') and β-actin (forward 5'-AGCGTGGCTACAGCTTCACC-3', reverse 5'-AAGTCTAGGGCAACATAGCACAGC-3'). PCR was carried out using Thermal Cycler 9600 (Perkin Elmer, Waltham, MA), annealing temperatures for BDNF and NT-3 primers were 60°C and 62°C and PCR cycle numbers were 32 and 35 cycles, respectively. All PCR products were separated on 1.2% agarose gels and the resulting bands for each sample were photographed by Lumibis Gel documentation (BioAmerica Inc., Miami, FL). All results were analyzed using Image J software (1.37 v, National Institutes of Health, Bethesda, MD), and the amounts of BDNF and NT-3 mRNA were corrected with β-actin. The results were shown as mean ± standard deviation of arbitrary unit.

### Immunohistochemistry

Upon completion of the urodynamic study, rats (n = 3 in each group and each experimental period) were deeply anesthetized and transcardially perfused with 150 ml of saline, followed by 500 ml of 4% paraformaldehyde in 0.12 M Phosphate Buffered Saline (PBS) via peristaltic pump. The thoracic spinal cord was removed, postfixed in 4% PBS for 4 hours and cryoprotected in 30% sucrose solution at 4°C for 5 days. Extracted tissues were identified and embedded in M1 embedding matrix (Thermo Fisher Scientific Inc., Waltham, MA) and kept at -80°C. Embedded spinal cords were cut sagittally at 20 μm using a cryocut microtome and then mounted on glass slides (Superfrost*, Thermo Fisher Scientific Inc., Waltham, MA). Previously mounted sections were treated with 0.2% Triton in 2% BSA/PBS solution for 5 minutes, washed and blocked with 10% normal serum for 1 hour. Primary antibodies diluted in 2% BSA/PBS solution were incubated for a day at 4°C and washed three times in 0.1 M PBS. Primary antibodies for immunohistochemistry were as follows: mouse anti-human nucleus (HN) antibody (1:200, Millipore Corp., Billerica, MA) for the detection of transplanted human cells, rabbit anti-glial fibrillary acidic protein (GFAP) polyclonal antibody (1:1000, Millipore Corp., Billerica, MA) for astrocytes, rabbit anti-beta III tubulin polyclonal antibody (1:1000, Covance, Emeryville, CA) for neurons and mouse ED1 monoclonal antibody (1:400, Millipore Corp., Billerica, MA) for macrophages and monocytes. Then secondary antibodies diluted in 2% BSA/PBS solution were incubated for 1 hour at room temperature and washed for three times. Secondary antibodies included FITC-conjugated goat anti-mouse IgG (1:200, Jackson Immunoresearch Labs, Inc., West Grove, PA) for monoclonal primary antibodies, and Rhodamine-conjugated goat anti-rabbit IgG (1:200, ICN Pharmaceuticals, Aurora, OH) for polyclonal primary antibodies. The sections were coverslipped using Vectashield* (Vector Laboratories, Inc., Burlingame, CA) and observed using fluoromicroscopy (Olympus, Tokyo, Japan).

For quantitation of HN and ED1 positive cells, digital image analysis using Image J software (1.37 v, National Institutes of Health, Bethesda, MD) was performed to count the number of HN and ED1 positive cells in the midline of sagittal section of injured spinal cord (n = 3) of all three groups at PTD28 and PTD56.

### Statistical analysis

Statistical analyses were performed using SPSS 15.0 (SPSS Inc, Chicago, IL). Differences in locomotor functions including BBB scale and coupling and ladder scores between experimental and control groups were examined for statistical significance using one-way analysis of variance (ANOVA) and Bonferroni's method for post-hoc comparison at each experimental period, and repeated measures ANOVA was performed to check whether the locomotor parameters were different between PTD28 and PTD56 groups in each hMSC and two control groups.

Differences of bladder volume and numeric parameters of urodynamic study between hMSC and control groups at PTD28 and PTD56 were analyzed using one-way ANOVA and Bonferroni's method for post-hoc comparison, and nominal data of urodynamic study between hMSC and control groups at PTD28 and PTD56 were compared using Fisher's exact test. Kruskal-Wallis test was performed to delineate any differences of BDNF and NT-3 levels between hMSC and control groups at PTD28 and PTD56.

For comparison of quantitative data of HN positive cells in hMSC and hFb groups and ED1 positive cells in hMSC and two control groups, Mann-Whitney U test and one-way ANOVA with Bonferroni post hoc test were used respectively. A value of p < 0.05 was considered significant.

## Authors' contributions

WBP, JP and JKH contributed to the design of the experiments; WBP, SHL, HWK and SYK contributed in vivo model making, transplantation of stem cells and fibroblasts, hindlimb function tests, and urodynamic study; JP contributed culture and preparation of stem cells and fibroblasts, ELISA and RT-PCR; JKH contributed immunohistochemistry and statistical analysis, and was involved in the writing of the manuscript. All authors read, corrected and approved the final manuscript.
